# Can public service motivation increase work engagement?—A meta-analysis across cultures

**DOI:** 10.3389/fpsyg.2022.1060941

**Published:** 2023-01-11

**Authors:** Mengxiao Ding, Chengli Wang

**Affiliations:** School of Public Policy and Management, China University of Mining and Technology, Xuzhou, China

**Keywords:** public service motivation, work engagement, civil servants, meta-analysis, cultural dimensions

## Abstract

Civil servants' work engagement is an essential topic in human resource management research of public sector. To explore the effects of public service motivation on civil service engagement as well as its mechanisms of action, and boundary conditions, this paper utilizes a meta-analytic approach to analyze 31 independent samples from 10 countries through literature search, screening, and coding. The result shows a significant positive relationship between public service motivation and work engagement with no possibility of publication bias. The regulatory effect test through Hofstede's model reveals that the dimensions of Power Distance Index, Individualism/Collectivism, Long-Term Orientation/Short-Term Orientation, and Indulgence/Restraint can significantly moderate the relationship between public service motivation and work engagement. This study provides a clear explanation for understanding the relationship between public service motivation and work engagement from a cross-cultural perspective, meanwhile it offers some theoretical implications for improving public servants' work engagement in the future.

## 1. Introduction

Work engagement, as a positive, fulfilling, work-related state of mind (Bakker et al., 2007), has received widespread attention in both public and private management (Schaufeli et al., 2008; Vigoda Gadot et al., 2013; Mostafa and Abed El-Motalib, 2020; Jeong et al., 2022). The civil servants, are public affairs managers and policies implementers, whose work engagement is closely related to the administrative efficiency, public services quality, and the government's image (Borst et al., 2017). Since the issue of work engagement has received significant attention in management, a lot of research has been conducted on how to improve individual work engagement (Eldor and Harpaz, 2019; Kwon and Kim, 2020; Tioumagneng and Njifen, 2020). The studies on civil servants' work engagement from the perspective of social psychology and organizational behavior are also gradually emerging in human resource management in the public sector (Christopher and Guy, 2004; Dawes et al., 2015; Arcangeli et al., 2018; Borst, 2018). Regarding the antecedents of work engagement, they have been mainly studied from the individual level and the organizational level (Harter et al., 2002; Bailey et al., 2017). Affective commitment, public service motivation, self-efficacy, emotional stability, personal initiative, and adaptability are vital personal factors that are closely correlated with high work engagement (Luu, 2018; Akingbola and Van Den Berg, 2019; Borst et al., 2020). From the organizational level, work engagement is closely related to the organizational sense of fairness, compensation and recognition, social support, job stress, leadership style and human resource management strategy (Bakker and Geurts, 2004; Schaufeli et al., 2009; Demerouti and Bakker, 2011). Among them, public service motivation is defined as “the beliefs, values and attitudes that beyond private and organizational interest, that concern the interest of a larger political entity and which induce through public interaction motivation for targeted action” (Vandenabeele, 2007). It is an essential individual factor in predicting and explaining the work engagement of public employees (Andersen, 2009) and is also crucial for public sector service and management (Homberg et al., 2017).

Increasingly, it is argued that civil servants' work engagement stems more from their intrinsic motivational factors, such as the pleasure, value, and meaning of work. These factors have a more decisive contribution to the civil servants' dedication than a rigid personnel system (Frank and Lewis, 2004). Public service motivation is an altruistic motivation to serve the interest of a group, a nation, or even humanity (Rainey and Steinbauer, 1999), which can motivate people to commit to public service unities and continuously increase their work enthusiasm (Perry et al., 2010). Public service motivation, as a psychological disposition to serve the public interest, can directly impact civil servants' work behavior (Andersen, 2009). It has been demonstrated that public service motivation can predict civil servants' job performance (Lynggaard et al., 2018), job satisfaction (Andersen and Kjeldsen, 2013), innovative behavior (Lee et al., 2020), and organizational citizenship behavior (Abdelmotaleb and Saha, 2018). Work engagement as a working status of individual, which is also influenced by public service motivation (Mendez and Avellaneda, 2022). Individuals with high levels of public service motivation have a clearer understanding of their responsibilities and missions. They are more likely to be motivated by the significance and value of their work, so they are more likely to mobilize their work resources and optimize their work needs, resulting in higher work engagement (Bakker, 2015; Jensen et al., 2018).

Extensive research has been conducted to reveal the link between public service motivation and work engagement. Some scholars view public service motivation as an individual's job resource that directly influences work engagement (Borst, 2018; Borst et al., 2020); some view public service motivation as a moderating variable between job resource and public service motivation (Bakker, 2015; Tensay and Singh, 2020). In general, the link between public service motivation and work engagement have has been largely confirmed. However, public service motivation is a dimensional construct, and different dimensions may relate differently to work engagement (Vinarski Peretz, 2020). Since individuals' public service motivation can be attributable to rational, normative, and affective motivation, clarifying the relative importance of various factors is more helpful in understanding the mechanism of action between public service motivation and work engagement (Taylor, 2007). Moreover, more needs to be done to explore of how the strength of the relationship between them varies across cultural contexts (Kjeldsen, 2014).

Meta-analysis allows for a comprehensive collection of relevant research literature, followed by quantitative synthesis to integrate independent research findings, thus getting a more general and accurate result from the macro perspective (Bangert-Drowns, 1986). This paper uses a meta-analysis to analyze the relationship between public service motivation and engagement for several reasons. First, the relationship between public service motivation and work engagement has been researched, and some scholars have focused on the correlation between subdimensions of public service motivation and work engagement (Yan et al., 2012; Zhu and Wu, 2016; Fang et al., 2020), which provides material for us to assess the relationship using meta-analysis. Second, there is some controversy about the strength and direction of the relationship between public service motivation and work engagement. Some scholars advocate that there is a medium positive correlation between them (Sun and Gu, 2017; Shim et al., 2021), but others hold different views (Eldor and Harpaz, 2019; Bland et al., 2021; Bashir et al., 2022). This paper aims to confirm the degree of the relationship by integrating existing studies. Finally, most current studies explore the relationship between public service motivation and work engagement mainly within a single country, without considering the cultural differences across countries. Indeed, cultural values not only influence individuals' attitudes, behaviors, and socialization (Kim, 2017), but also the structure and meaning of public service motivations (Harari et al., 2016). Therefore, this paper explores the differences in the strength of the relationship between public service motivation and work engagement from a cross-cultural perspective.

The possible contributions of this study are as follows. First, the correlation between public service motivation and work engagement is validated by means of a meta-analysis, which helps to clarify previous research controversies regarding relationship strength. Second, analyzing the relationship between different dimensions of public service motivation and work engagement helps to understand more deeply the dimensional differences and effect of different dimensions. Finally, with the cultural dimensions as moderators to examine the relationship differences between public service motivation and work engagement, this study provides a cross-cultural perspective for understanding the boundary condition of this link. It will also provide a theoretical guide for management practices in the public sector that reinforce national cultural values.

## 2. Literature review and hypothesis

### 2.1. Public service motivation

The theory of public service motivation originated from the criticism and reflection on the hypothesis of the “economic man” in the public sector. Under the influence of the “economic man” hypothesis, people often believe that the main ways to motivate public sector personnel are salary and status (Crewson, 1997). Whereas, Rainey (1982) found that compared with private sector workers, public sector employees are less motivated by material and status pursuits. Public service motivation refers to “an individual's predisposition to respond to motives grounded primarily or uniquely in public institutions and organizations” (Perry and Wise, 1990). It contains three components: rational motivation, normative motivation, and affective motivation. Based on this, Perry (1996) proposed a public service motivation measure scale with 24 items covering four dimensions: attraction to policy making (APP), commitment to the public interest (CPI), compassion (COM), and self-sacrifice (SS). And this four-dimensional scale has been widely used internationally. Among them, the rational motivation that drives individuals to engage in public service comes from the opportunity to participate in public policy making, which is excessively attractive to people and can enhance their sense of value to contribute to society and serve the public. The normative motivation comes from the commitment to the public interest, which is individual's altruistic behavior and reflects the individual's sense of responsibility and loyalty to the public. The emotional motivation to engage in public service primarily stems from their compassion for others and self-sacrifice, which is the behavioral motivation for individuals to respond emotionally to social situations (Perry, 2000).

The concept and measurement of public service motivation are not universally accepted due to the different cultural scenarios, and scholars have begun to re-examine its vision and operational definition (Cerase and Farinella, 2009; Giauque et al., 2011). Some scholars argued that the measurement should be appropriately abridged (Leisink and Steijn, 2009) or combined (Vandenabeele, 2008; Ritz, 2011) according to specific cultural differences. For example, Liu et al. (2008) argued that the dimension of “compassion” is not applicable in China. Kim et al. (2013) developed and tested Perry's scale in 12 countries and modified it according to the culture, language, and values of different countries, and proposed a universal cross-cultural international scale covering four dimensions: attraction to public participation, commitment to public values, self-sacrifice, and compassion, and streamlined the questions into 16 items. A growing number of scholars have also argued that public service motivation is an altruistic motivation to serve the community or other public interests, thus advocating extract 5 items from Perry's initial development scale for a global measure of public service motivation (Wright et al., 2013). In summary, based on previous research, this paper classifies the measurements for public service motivation as composite and global measures (Ironson et al., 1989; Min et al., 2021).

### 2.2. Work engagement

Work engagement refers to a positive and substantial psychological state related to work which is characterized by vitality, dedication, and concentration (Lawler and Hall, 1970). Employees with higher levels of work engagement tend to have more positive emotions and better mental and physical health (Luu, 2019). This concept was first introduced by Kahn (1990), who claimed that work engagement consisted of physical, cognitive, and emotional engagement. This imply that employees are able to maintain a high state of physiological arousal, cognitive activation, and emotional sensitivity while performing the role tasks assigned to them by their jobs (May et al., 2004). In contrast to job burnout, work engagement is characterized by energy, job involvement, and high efficiency. Employees with high work engagement can devote themselves to work with full energy and spirit and believe that they can be fully competent for their work (Drory and Shamir, 1988). Schaufeli (2002) considerd work engagement to be composed of three components: vigor, dedication, and absorption. Based on this, he developed the Utrecht Work Engagement Scale (UWES). At first, the scale had 17 questions in total. Afterwards, Schaufeli et al. (2006) reduced the scales to 9 questions, which has been widely used. Schaufeli et al. (2008) believed that the word “work engagement” should be replaced by “dedication” because dedication is more in-depth than work engagement in terms of quantity and quality, which means that employees have a strong recognition of work in terms of cognition and emotion. In addition, Britt et al. (2001) developed a work engagement measurement scale containing three dimensions, namely, responsibility, commitment, and performance impact perceptions. Gallup developed the GWA scale to measure employee attitudes and factors affecting employee attitudes, with 12 questions. In general, regarding the measurement of work engagement, scholars have formulated different scales with unusual dimensions from specific definitions, but relatively speaking, the UWES scale is more widely used.

### 2.3. The relationship between public service motivation and work engagement

It has been found that public service motivation plays a pivotal role in providing employee engagement as a psychological resource (Gross et al., 2019). Public service motivation affects how public sector employees handle the demands and resources of their daily work. As early as 1975, Buchanan (1975) studied the relationship between public service motivation and work engagement, noting that solid expectations of loyalty and dedication to the organization were essential characteristics of public service, which led to significantly higher levels of work engagement among public managers than among business managers.

Public service motivation is often seen as a direct or indirect predictor of work engagement. Scholars have explored their relationship from the perspectives of Self-Determination theory, Person-Environment Fit theory, and Job Demands-Resources theory. Self-Determination theory suggests that employees' perceived intrinsic work motivation is the cornerstone of higher work engagement (Ryan and Deci, 2000; Gagné and Deci, 2005). According to Person-Environment Fit theory, public service motivation is an individual personality factor influenced by the degree to which these personality qualities are supported and nurtured in the organizational environment, indirectly impacting work engagement (Boyd and Nowell, 2020). However, the Job Demands-Resources (JD-R) model argues that there is an implied interaction between demands and resources that can more fully define the relationship between public service motivation and work engagement (Borst et al., 2017). According to this model, the specific risk factors associated with job stress include two broad categories: job demands and resources. Job demands refer to the negative factors that consume individual energy, such as work overload, role ambiguity, task complexity, time pressure, and insecurity (Bakker, 2015), which can weaken the individual's work engagement. Job resources are the positive factors that help employees deal with work demands, including psychological resources, development opportunities, and organizational support (Bakker and Demerouti, 2007), which are beneficial to improve the individual's work engagement.

According to the JD-R theory, civil servants with high levels of public service motivation can actively regulate their work resources, such as social support from colleagues, and performance feedback, to remain dedicated and achieve good performance (Mussagulova, 2021). That is to say, those with high levels of public service motivation can make better use of their surrounding resources, thus presenting high work engagement. JD-R theory proposes that employees have personal resources to help them handle job demands. Compared with job resources, personal resources are self-beliefs that possess resilience and motivation, which can help individuals overcome the difficulties they encounter at work (Hobfoll et al., 2003). Personal resources such as optimism, self-efficacy, and substantial self-esteem help motivate individuals to be more engaged and contribute to the organization beyond the demands and resources of the job (Xanthopoulou et al., 2009). Some scholars also believe that public service motivation can have a buffering effect between job demands and work engagement. It can weaken the negative impact of job demands, such as red tape on work engagement, and balance the relationship between job demands and job resources, thus allowing individuals to maintain a high level of work engagement (Cooke et al., 2019).

**Figure 1:**
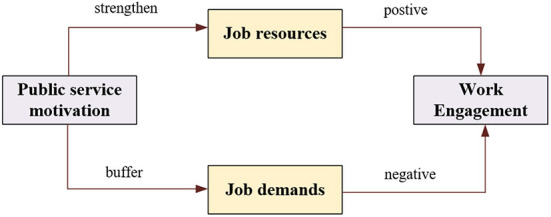
The complete JD-R model of the relationship between public service motivation and work engagement (public service motivation can strengthen the positive effect between work resources and engagement, while buffering the adverse effects between work demands and work engagement).

To summarize, the influence of public service motivation on work engagement is mainly manifested in the following aspects. First, public service motivation strengthens the link between work resources and work engagement, which can motivate individuals to allocate their work resources rationally and thoroughly mobilize the resources around them, thus improving their work engagement. Second, public service motivation buffers the adverse effects between work demands and work engagement (Abdelmotaleb, 2020). Individuals with higher public service motivation possess optimistic, positive psychological states and pro-social motivation. They are less affected by environmental stress and their own stress, which in turn weakens the relationship between emotional exhaustion and depersonalization, and guarantees higher work engagement (Mussagulova, 2021) (the complete JD-R model of the relationship between public service motivation and work engagement is depicted in Figure 1). Therefore, this paper proposes,

H1: There is a positive correlation between public service motivation (attractiveness of public policy, commitment to public interest, compassion, and self-sacrifice) of civil servants and their work engagement.

### 2.4. Cultural dimensions

National culture is a core organizational principle for how employees understand their work, how they treat it, and how they expect to be treated. Although little had been done on the degree of the correlation between public service motivation and work engagement in terms of Hofstede's cultural dimension theory, the role of culture has been recognized. Sun and Gu (2017) argued that under the influence of high power distance, an individual's motivation for public service may have less to do with an individual's desire for the public good or altruism and more with personal or family motivation. Bashir et al. (2022) pointed out the need to explore the impact of public service motivation on the attitudes and behaviors of public employees from the perspective of cultural context. As is revealed by their study, the effect of public service motivation on work engagement is more remarkable when individuals' perceptions of social justice are low. Moynihan and Pandey (2007) found that work engagement was significantly related to public service motivation, group culture, and promotion opportunities. However, scholars have mainly explored the applicable scenarios of public service motivation and the relationship between public service motivation and work engagement from a single cultural dimension. Therefore, by drawing on Hofstede's cultural dimension theory, this paper provides an in-depth analysis of the relationship between public service motivation and work engagement from six cultural dimensions in order to contour the boundary conditions of the relationship between them.

Hofstede (1984) regarded national culture as a collective project that distinguished one group from another. He classified ethnic culture into six dimensions: Power Distance (PDI), Individualism/Collectivism (IDV), Masculinity/Femininity (MAS), Uncertainty Avoidance Index (UAI), Long-Term Orientation/Short-Term Orientation (LTO), and Indulgence /Restraint (IVR) (Hofstede, 2006).

Power Distance is the acceptance of unequal distribution of power in a society or organization by those in a disadvantaged position (House et al., 2004). Acceptance of inequality varies across cultures due to differences in how power distance is interpreted differently. In a low power distance culture, individuals enjoy equal power. They have higher participation within the unitary organization (Karen and Stanley, 1996), which is more helpful for individuals to translate their public service motivations into concrete work engagement. Therefore, this paper proposes,

H2: Power distance Index moderates the relationship between public service motivation and work engagement, and the relationship is more robust in the low power distance context.

Individualism/Collectivism is a dimension that implies whether society is more concerned with individual or collective interests. Public service motivation aligns with collectivist rather than individualist norm because collectivist culture emphasize communal value and interest. In collectivist culture, people perceive themselves as belonging to a group and thus act according to their group's interest, which are not always aligned with their interest (De Dreu et al., 2000). In the context of a collectivist culture, it is also appropriate for individuals to sacrifice themselves for the common interest and generate higher levels of work engagement. Therefore, this paper proposes,

H3: Individualism/Collectivism moderates the relationship between public service motivation and work engagement. And the relationship is more robust in the context of collectivism.

Masculinity/Femininity refers to whether a society represents more masculine qualities such as competitiveness and assertiveness or more feminine qualities such as tolerance and modesty (Hofstede, 1991). The importance that work occupies in a person's life can vary depending on cultural differences in masculinity. In a country where women are valued, and gender equality is pursued, individuals are more concerned about others and the interpersonal relationships around them (Singh and Mohanty, 2011). As a result, individuals are more likely to translate their public service motivation into work engagement. Therefore, the following hypothesis is proposed,

H4: Masculinity/Femininity moderates the relationship between public service motivation and work engagement. And the relationship is more robust in the context of femininity.

Uncertainty Avoidance Index refers to the degree to which society tolerates uncertainty and ambiguity, with different attitudes toward the perception of uncertainty across cultures (Hofstede and Mcrae, 2004). In government institutions, uncertainty is expressed in the clarity of plans, rules, legal texts, and procedures. In weak uncertainty-averse societies, civil servants are optimistic about the political process. People trust politicians and the judicial system, and trust mechanisms in society are high, which is conducive to making civil servants generate higher work engagement. Therefore, it is hypothesized,

H5: Uncertainty Avoidance Index moderates the relationship between public service motivation and work engagement. Moreover, the relationship is more robust in the context of a weak uncertainty avoidance culture.

Long-Term Orientation/Short-Term Orientation refers to the extent to which society is comfortable with delaying the satisfaction of its material, emotional, and social needs (Hofstede et al., 2010). A culture of long-term orientation is characterized by patience, perseverance, submission, and accountability for the greater welfare. In a long-term orientation culture context, people are more satisfied with their contribution to eliminating inequality and injustice in society and bringing about a fair and equal life for everyone (Clugston et al., 2000). In contrast, in a short-term orientation cultural context, people have difficulty being satisfied with their efforts to achieve public profit. Therefore, this paper proposes,

H6: Long-term orientation/Short-term orientation moderates the relationship between public service motivation and work engagement, and the relationship is more robust in the long-term orientation cultural context.

Indulgence/Restraint refers to the extent to which basic needs and enjoyment of life are allowed. The culture of restraint places less emphasis on personal recreation and desire fulfillment than indulgence (Hofstede et al., 2010). Individuals under the influence of this perception are bound by social norms in their behavior and believe that it is wrong to indulge themselves. People have different values toward personal pleasures and desires that affect their work engagement. Therefore, it is hypothesized,

H7: Indulgence/Restraint moderates the relationship between public service motivation and work engagement. And the relationship is more robust in cultures that advocate self-restraint.

## 3. Methods

Meta-analysis is a systematic method of quantitative generalization and summarization of empirical research findings, which can avoid sampling and measurement errors to the greatest extent and present the relationship between variables as realistically as possible (Hunter and Schmidt, 2004). It has the advantage of clarifying previous research divergence and obtaining more convincing conclusions (Moreau and Gamble, 2020).

### 3.1. Literature search

**Figure 2:**
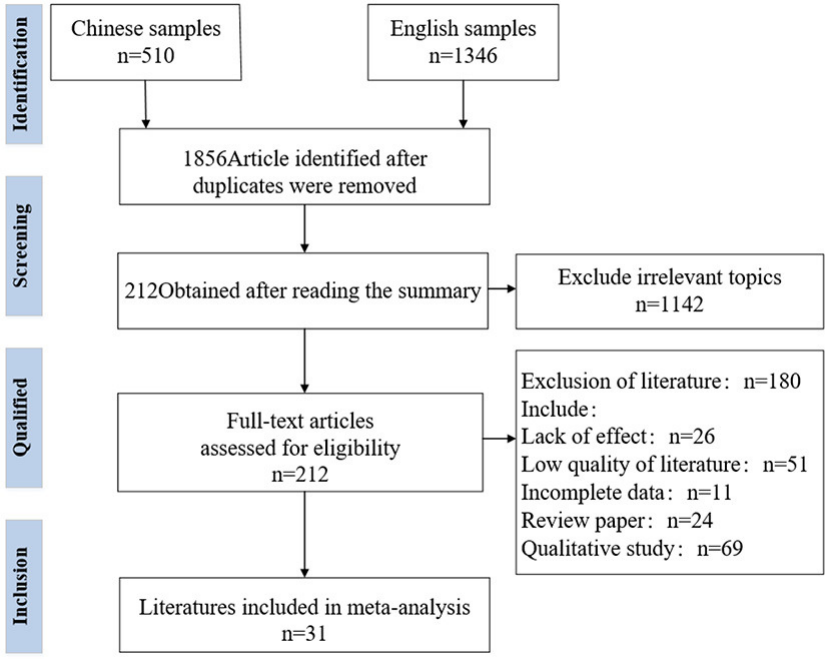
PRISMA literature screening diagram (according to the inclusion criteria, we finally got 31 pieces of literature related to our research to make a meta-analysis).

Literature search was conducted with web of science, EBSCO, Springer, Scopus, Google Scholar databases, Zhiwang, Google Scholar, and Baidu Scholar in both English and Chinese. The search terms were limited to “public service motivation or PSM” and “work engagement.” As of July 17, 2022, 1,856 documents were obtained, including 510 in Chinese and 1,346 in English. To ensure the relevance of the literature, the abstracts and titles of 1,856 articles were imported into NoteExpress for checking and then preliminary screening was done to obtain 212 articles matching the theme of “public service motivation and work engagement.” Figure 2 reports the literature search results of this study.

### 3.2. Inclusion criteria

This paper follows the following inclusion criteria: First, it had to be a quantitative study, excluding the qualitative research literature. Second, the research literature has clearly stated the total sample size and the r value of the correlation coefficient between public service motivation and work engagement, or the *F*-value, *t*-value, and χ^2^ that could be translated into a correlation coefficient. Third, the research subjects must be groups engaged in public services, such as civil servants, police officers, teachers, and other practitioners of public nature, and the country or region to which the sample belongs must be indicated. Fourth, only one of the data from the same author will be used so as to avoid repeated use.

The specific literature screening flow chart is shown in Figure 2. Based on the above criteria, 31 independent samples were finally obtained from 10 countries involving 70,918 research respondents. Three of these pieces of literature only reported the correlation coefficients between the four dimensions of public service motivation and work engagement, instead of the total correlation coefficients.

### 3.3. Coding procedures

The 31 documents included in the analysis were coded and then proofread to ensure consistency and objectivity of coding. The coding information included “author + publication date,” “correlation coefficient,” “sample size,” “PSM measurement tool (composite or global),” and “WE measurement tool (UWES or other),” “country,” and cultural dimensions (including PDI, IDV, MAS, UAI, LTO, IVR). The information on cultural dimensions is judged according to the country of the research object involved in the literature and Hofstede's cultural dimension survey score of 112 countries (specific information comes from: http://www.geert-hofstede.com/). In addition, since three of the literature only report the correlation coefficients between the dimensions of public service motivation and work engagement, they were coded separately according to their four dimensions (APP, CPI, COM, and SS). The specific coding content can refer to Appendix.

### 3.4. Statistical analysis

The software Comprehensive Meta-Analysis Version 3.0 was used to estimate the main effect and test for moderating effects on the correlation coefficients (r) included in the analysis. Fisher was used to convert r into approximately normally distributed Zr values and then into correlation coefficients to present the results (Ringquist, 2013). The specific calculation process is as follows.

Firstly, the correlation coefficient *r* is converted to Fisher's *z*-value according to the formula:


$$\begin{array}{l} Z_{i}=0.5*\ln\left[\left(1+r_{i}\right)/\left(1-r_{i}\right)\right] \end{array}$$

Then it is weighted according to the size of the study sample.


$$\bar{Z}=\frac{\sum N_{i}Z_{i}}{\sum N_{i}}$$

Finally, *Z* is converted into a correlation coefficient:


$${\text{r}}_{\text{z}}=({\text{e}}^{\text{2}\bar{\text{z}}}-1)/({\text{e}}^{\text{2}\bar{\text{z}}}+1)$$

## 4. Results

### 4.1. Heterogeneity test

The heterogeneity test, also known as the consistency test of statistics, aims to check whether the results of independent studies can be combined. The *Q*-value and *I*^2^ statistics are the main ways to detect the presence and the degree of heterogeneity (Rice, 2009). When the heterogeneity is significant, a random effects model is appropriate to correct for the combined effect values to make the results more accurate; if the heterogeneity is small, a fixed effects model is more appropriate. Heterogeneity test is reported in Table 1. A heterogeneity test of the effect values of the 28 samples included in the analysis revealed significant heterogeneity among the sample sizes (*Q* = 888.439 and *p* < 0.05). The *I*^2^ statistic of 96.961% implies high heterogeneity among the effect values regarding the relationship between public service motivation and work engagement, and only 3.039% of the variance is caused by sampling (Higgins et al., 2002). Therefore, further moderating effect tests are needed to determine the source of heterogeneity. In summary, through the heterogeneity test, it is found that there are significant differences between the effects included in the analysis. Accordingly, it is suitable to use the random effect model for meta-analysis.

**Table 1:** Heterogeneity test results (the *I*^2^ statistic of 96.961% implies high heterogeneity among the effect values, therefore, random effect model is suitable for this meta-analysis).

**Model**	**Number studies**	**Effect size and 95% interval**			**Test of null (2-Tail)**		**Heterogeneity**			
		**PE**	**LL**	**UL**	**Z**	***P***	**Q**	**df**	***P***	*I* ^2^
Fixed	28	0.438	0.431	0.446	100.293	0.000	888.439	27	0.000	96.961
Random	28	0.359	0.306	0.410	12.232	0.000				

PE, Point Estimate; LL, Lower Limit; UL, Upper Limit; Z, Extent of deviation from variance; P, Testing the likelihood of the null hypothesis holding; Q, Statistical test used for the estimation of heterogeneity; df, Degree of freedom; I^2^, Proportion of effect size variance that can be attributed to moderator variables (%).

### 4.2. Publication bias test

Previous research has shown that published studies have larger mean effect values than unpublished studies, making it particularly important to conduct publication bias tests on the sample of studies included in the analysis (Thornton and Lee, 2000). The meta-analysis of the relationship between public service motivation and work engagement in this paper did not involve unpublished literature, which may affect the reliability of the meta-analysis results. Therefore, three methods, namely, funnel plot, Egger's regression coefficient, and Fail-safe N, were used to conduct the publication bias test.

**Figure 3:**
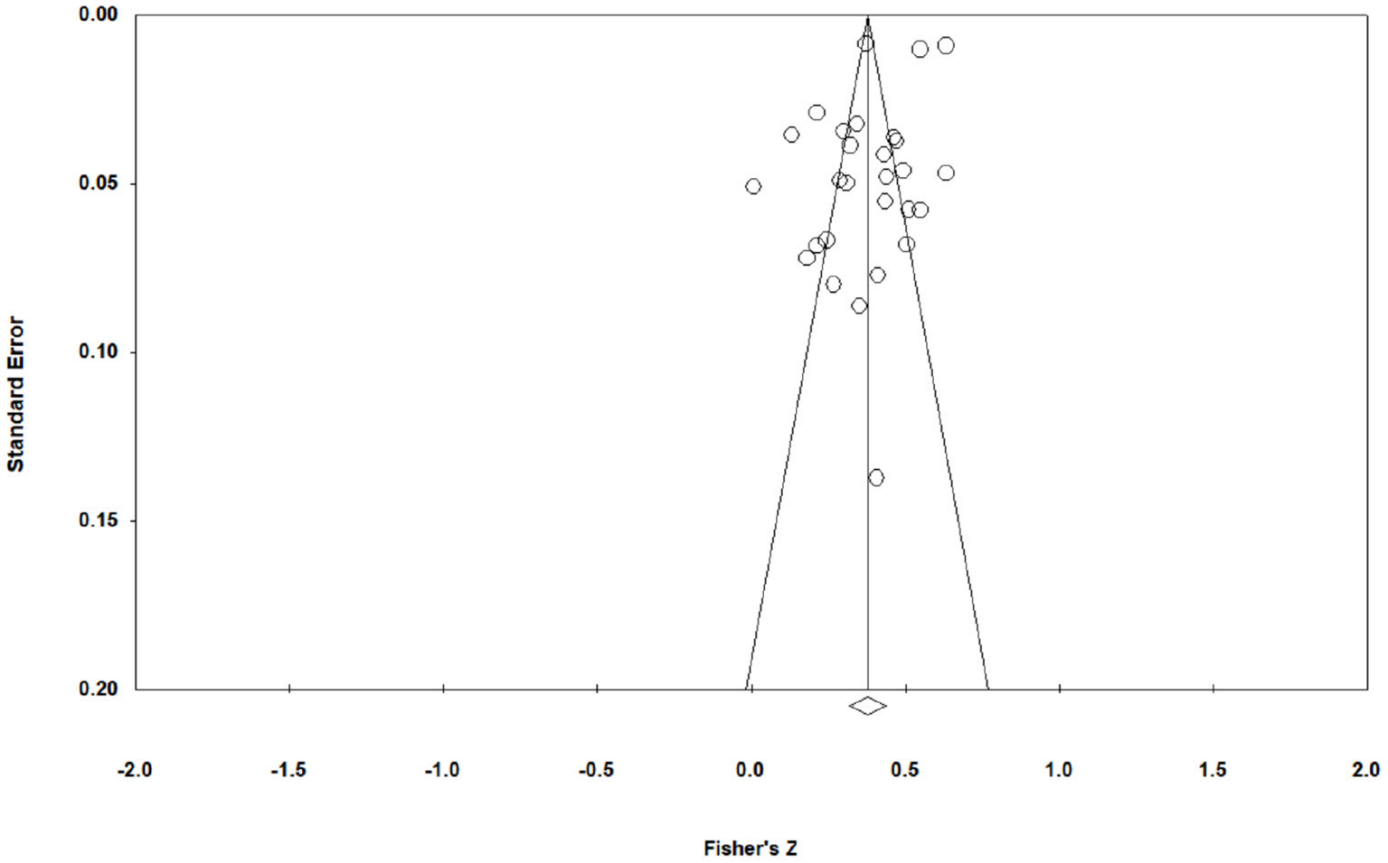
Funnel plot of the distribution of each effect value (the effect values of public service motivation and work engagement are mainly concentrated at the top of the funnel plot and spread more evenly on both sides of the midline 0.359).

One of the earliest methods for identifying publication bias, and still among the most popular, is the funnel plot. Generally, when the points represented by each effect value are concentrated at the top of the funnel plot, and the curve spreads downward and evenly on both sides of the midline, it proves that there is no publication bias. Conversely, it proves that there exits publication bias (Kepes et al., 2012). From Figure 3, which displays the funnel plot of the distribution of each effect value, it can be seen that the effect values of public service motivation and work engagement are mainly concentrated at the top of the funnel plot and spread more evenly on both sides of the midline 0.359. Accordingly, it can be tentatively judged that there is less possibility of publication bias in this study.

However, the test results of the funnel plot are relatively subjective. In order to further ensure the accuracy of the study results, this paper uses Egger's regression coefficient test for in-depth verification (Song and Gilbody, 1998). As can be seen from Table 2, the result of Egger's test is intercept = −3.430 and *P* < 0.05, which proves the possibility of publication bias.

**Table 2:** Egger's regression coefficient test and classic fail-safe *N* test results (these tests, along with the funnel pot are to determine if there is publication bias).

**Variable**	**Egger's regression intercept**					**Classic fail-safe** ***N***		
	**Intercept**	**SE**	***t***	**df**	***P***	**Z**	***P***	***N***
PSM	−3.430	1.471	2.331	26	0.027	64.791	0.000	10,570

SE, Standard error of the average effect size; df, degree of freedom; P, Testing the likelihood of the null hypothesis holding; Z, Extent of deviation from variance; N, Number of missing studies that would bring p-value to >alpha.

This paper uses Fail-safe N validation to measure the severity of publication bias. This method assesses how many unpublished studies (N) are needed to make the total effect size of published studies insignificant. If N is much larger than 5k + 10 (k = published sample size), the results of the meta-analysis are shown to be insensitive to publication bias (Rosenthal, 1979). As can be seen from the Table 2, the Fail-safe coefficient for the study on public service motivation and work engagement was 10,570, which is well above the critical value of 150. In summary, the final test showed that although there is slight publication bias, the bias is within the acceptance range, and the results of the meta-analysis were reliable.

### 4.3. Main effect test

Based on the above heterogeneity test results, this paper analyzes the correlation between public service motivation and its dimensions with work engagement using a random effect model. As shown in Table 3, the correlation coefficient between public service motivation and work engagement is 0.359 with *p* < 0.001. According to the judgment of Arya et al. (2020), the r values of 0.10, 0.25, and 0.40 represent a low, medium, and a high degree of correlation, respectively. Thus, it can be concluded that there is a medium positive correlation between public service motivation and work engagement. Sensitivity analysis of the effect sizes showed that after arbitrarily excluding one sample, the correlation coefficients of them still fluctuated between 0.306 and 0.410, indicating high stability of the effect sizes.

**Table 3:** Main effects test (this table reported the relationship between public service motivation and its subdimensions with work engagement).

**Variables**	**K**	***N***	**PE**	**LL**	**UL**	**Z**	***P***
PSM	28	71,606	0.359^***^	0.306	0.410	12.232	0.000
APP	4	26,070	0.288^***^	0.220	0.353	8.040	0.000
CPI	4	26,070	0.385^***^	0.365	0.404	34.733	0.000
COM	4	26,070	0.353^***^	0.234	0.462	5.523	0.000
SS	3	1,736	0.501^***^	0.291	0.666	4.289	0.000

k, Sample size; N, Research respondents; PSM, Public service motivation; APP, Attraction to policymaking; CPI, Commitment to public interest; COM, Compassion; SS, Self-sacrifice; PE, Point Estimate; LL, Lower Limit; UL, Upper Limit;

*** , ^**, *^Indicate variables significant at the 0.001, 0.01, and 0.05 levels, respectively.

To further understand the differences in the relationship between subdimensions of public service motivation and work engagement, each subdimension was analyzed with a sample size (k) >3. Table 3 also shows that all four dimensions of public service motivation are all positively correlated with work engagement, with attraction to policy making (*r* = 0.288), commitment to the public interest (*r* = 0.385), and compassion (*r* = 0.353) having a medium positive relationship with engagement, and self-sacrifice (*r* = 0.501) having a high positive relationship with work engagement. In terms of significance level, all four dimensions are significant at the 0.001 level. Thus, H1 was confirmed.

### 4.4. Moderating effect test

Since different measurement instruments have specific contents and structures, this paper examines the moderating effects of the relationship between public service motivation and work engagement from the six cultural dimensions in addition to the public service motivation and the work engagement measurement instruments. The results are shown in Table 4. As can be seen, there is no significant difference in the relationship between public service motivation and work engagement using either the composite measure or the global measure of public service motivation (*Q* = 1.075, *p* = 0.300). Similarly, there is no significant difference in the relationship between public service motivation and work engagement using the UWES or other work engagement scales (*Q* = 0.737, *p* = 0.391).

**Table 4:** Results of the test for moderating effects (this table reported the results of the moderating effect tests for the measurement instruments and cultural dimensions).

**Moderator**	**Category**	**k**	***N***	**Effect size and 95% interval**			**Heterogeneity**		
				**PE**	**LL**	**UL**	***Q***	**df**	***P***
PSM measurement	Composite	16	30,506	0.340	0.261	0.414	1.075	1	0.300
	Global	12	16,552	0.388	0.337	0.436			
WE measurement	UWES	17	28,429	0.383	0.326	0.436	0.737	1	0.391
	Others	11	18,843	0.323	0.191	0.443			
PDI	Higher	19	19,823	0.404	0.335	0.468	5.717	1	0.017
	Lower	9	26,474	0.263	0.165	0.356			
IDV	Collectivism	18	19,686	0.407	0.336	0.473	5.718	1	0.017
	Individualism	10	26,611	0.269	0.176	0.358			
MAS	Higher	21	21,928	0.342	0.293	0.389	1.536	1	0.215
	Lower	7	24,369	0.407	0.315	0.491			
UAI	Higher	6	12,954	0.423	0.295	0.535	1.333	1	0.248
	Lower	22	33,343	0.343	0.289	0.394			
LTO	Long-term	18	28,822	0.413	0.359	0.465	11.165	1	0.001
	Short-term	10	17,475	0.256	0.177	0.331			
IVR	Higher	9	26,474	0.263	0.165	0.356	5.727	1	0.017
	Lower	19	19,823	0.404	0.335	0.468			

k, Sample size; N, Research respondents; WE, Work engagement; PSM, Public service motivation; PDI, Power Distance Index; IDV, Individualism/collectivism; MAS, Masculinity/femininity; UAI, Uncertainty Avoidance Index; LTO, Long-term orientation/Short-term orientation; IVR, Indulgence/Restraint; PE, Point Estimate; LL, Lower Limit; UL, Upper Limit.

The test for moderating effects of cultural dimensions is also shown in Table 4. As is shown, there is a significant difference in the effect of Power Distance Index (*Q* = 5.717, *Q* < 0.05), and the correlation between public service motivation and work engagement is significantly higher in the cultural context of high Power Distance Index (*r* = 0.468) than in the cultural context of low Power Distance Index (*r* = 0.356). Thus, the first half of H2 is verified, but the latter part of H2 assumes to be the opposite to the result. There is also a significant difference in the effect of Individualism/Collectivism on the relationship between public service motivation and work engagement (*Q* = 5.718, *p* < 0.05), with a significantly higher correlation in the cultural context of Collectivism (*r* = 0.473) than in the cultural context of Individualism (*r* = 0.358), thus confirming H3. There is no significant difference in the effect of Masculinity/Femininity (*Q* = 1.536, *p* > 0.05) and Uncertainty Avoidance Index (*Q* = 1.333, *p* > 0.05) on the relationship between public service motivation and work engagement, thus H4 and H5 are rejected. The index of Long-Term Orientation/Short-term Orientation has a significant effect on the relationship between public service motivation and work engagement, and the relationship is more robust in Long-Term Orientation (*r* = 0.465) than in Short-Term Orientation (*r* = 0.331). Accordingly, H6 is confirmed. The index of Indulgence/Restraint has a significant moderating effect on the relationship between public service motivation and work engagement, and the relationship is significantly lower in the context of pursuing self-indulgence (*r* = 0.356) than in the cultural context of pursuing self-restraint (*r* = 0.468). In this sense, H7 is confirmed, too.

## 5. Discussion

This paper verified the relationship between public service motivation, including its subdimensions, and work engagement through meta-analysis. Furthermore, the meta-analysis results showed no publication bias.

### 5.1. Main effects analysis of public service motivation and work engagement

In this paper, from the psychological perspective of public service motivation, a meta-analysis of 28 independent studies revealed a moderate positive relationship between individuals' public service motivation and work engagement. That is, individuals with higher levels of public service motivation also tend to have higher levels of work engagement, which is consistent with the previous research results (Geyfman, 2014; Jin and Mcdonald, 2017). Although the causal relationship between public service motivation and work engagement cannot be determined, accordingly, the results suggest that public service motivation has some value in improving work engagement, thus demonstrating the practical significance of this study. It is of great relevance to improve public service motivation of public employees to enhance their dedication and engagement to work.

The meta-analysis results confirm the explanatory power of the Job Demands-Resources theory (JD-R) on the relationship between public service motivation and work engagement (Van Loon et al., 2015). Job Demands-Resources theory suggests that a high degree of work engagement implies a high job demand level. In other words, individuals need physical, psychological, organizational environmental, and social support to maintain a high level of work engagement. Public service motivation is the intrinsic motivation that drives individuals to maintain the organization's interests and serve the public (Vandenabeele, 2007). By this motivation, individuals engaged in public service have a higher sense of mission, responsibility, and self-sacrifice, as well as a higher degree of recognition and self-worth for their work. Therefore, based on Job Demands-Resources theory, public service motivation is a kind of psychological energy and resource that can motivate individuals to overcome their dissatisfaction and complaints about work and lead to a high level of dedication. However, the results of the meta-analysis also show that the relationship between public service motivation and work engagement is just moderately correlated, implying that we should not overstate the role of public service motivation and ignore the role of various factors such as organizational support and social support for public sector workers.

There is also a positive correlation between all the dimensions of public service motivation and work engagement, and there are different degrees of relationships between the four dimensions of public service motivation and work engagement. Among them, self-sacrifice (SS) has the strongest correlation with work engagement, whereas attraction to public policy making (APP) has the weakest. These results also imply that individuals more committed to their work are more motivated by self-sacrifice and less by the attractiveness of policy making. In essence, self-sacrifice is a spirit of devotion that is willing to put personal interests and power aside for the collective interest, and it comes from the emotional motivation of the individual. The attraction to public policymaking (APP) is an individual rational motivation driven by the need for power and self-esteem (Van Loon et al., 2018). Furthermore, most public sector workers play more of an executive role in the day-to-day management of government affairs and less of a significant role in policy making. By contrast, the desire to do socially valuable work (normative motivation) and the willingness to help others (affective motivation) are the primary motivators of civil servants' work engagement (Bright, 2013). Therefore, this may be why the self-sacrifice (SS) dimension has a stronger relationship with work engagement. This finding also corroborates the assertion that the strength of the association between public service motivation and affective commitment, motivation, job engagement, and job satisfaction varies considerably depending on the subdimensions of public service motivation (Taylor, 2007; Homberg et al., 2015; Borst, 2018).

### 5.2. Moderating effects of measurement instruments and cultural dimensions

This paper focused on examining the potential moderating variables between public service motivation and work engagement from the perspectives of both measurement instruments and cultural dimensions. The final results show that neither the public service motivation nor the work engagement measurement instrument has a significant moderating effect on the relationship between them. However, this does not mean that we can blindly apply the public service motivation measurement scale and the work engagement measurement scale in the actual measurement. Instead, scenario-specific measurement tools should be developed by appropriately modifying the existing dimensions in the context of specific research scenarios.

From the perspective of cultural dimensions, Power Distance Index, Individualism/Collectivism, Long-Term Orientation/Short-Term Orientation, and Indulgence/Restraint have a significant moderating effect on the relationship between public service motivation and work engagement.

First, Power Distance Index significantly moderates the relationship between public service motivation and dedication. But contrary to the H2, the relationship between public service motivation and work engagement is stronger in cultures with high Power Distance Index and weaker in cultures with low Power Distance Index. This may be because public service motivation, as a psychological factor, is moderated by the individual's emotional state in terms of its impact on actual behavior (Wright and Bonett, 2007). In high Power Distance contexts, public sector workers are more tolerant of inequities in their organizations. When negative behaviors such as income inequality and corruption occur in society, individuals legitimize them subjectively and psychologically and thus generate fewer negative emotions (Auh et al., 2016). On the contrary, in a culture with low Power Distance, individuals are more likely to be stimulated by some unjust events and to develop negative slackness. Work attitude and work emotion are essential factors influencing organizational members' work engagement. Accordingly, so in a high Power Distance context, individuals' motivation for public service is also more likely to be translated into a commitment to work engagement.

Second, Individualism/Collectivism significantly moderates the relationship between public service motivation and work engagement. The findings reveal that the relationship between public service motivation and work engagement is more robust in the collectivist cultures. In the collectivist cultural context, individuals within the unit pay more attention to achieving organizational goals and shaping good interpersonal relationships (Chen et al., 2015). In contrast, in the individualist cultural context, individuals pay more attention to their development and interests and strongly need autonomy and independence (Jackson et al., 2006). Thus, motivated by a collectivist culture, individuals possess a stronger spirit of self-sacrifice, a stronger sense of identification with the public interest, and a stronger sense of recognition and value for the effort they put into the collective. According to the Job Demands-Resources theory, when individuals feel support from the organization, they are also willing to give back to the organization with higher performance, leading to a stronger relationship between public service motivation and work engagement in a collectivist culture scenario.

Again, Long-Term Orientation/Short-Term Orientation significantly moderates the relationship between public service motivation and work engagement. As has been revealed by this study, the correlation between public service motivation and work engagement is robust in the Long-Term Orientation context. This result may be because, in long-term socially oriented situations, individuals have a more robust engagement to the future and long-term relationships and possess more perseverance and persistence (Schaufeli, 2018), thus making their public service motivation a stronger positive incentive for their work engagement. They are committed to public service with enthusiasm and loyalty, resulting in a higher level of engagement. On the contrary, a culture that tends to be more short-term oriented is more concerned with immediate benefits and more in pursuit of results, which may be the reason for the weaker relationship between public service motivation and work engagement.

Finally, Indulgence/Restraint significantly moderates the relationship between public service motivation and work engagement. The correlation between public service motivation and work engagement is weaker in a cultural scenario of self-indulgence and more substantial in a cultural scenario of self-restraint. In a society where indulgence is practiced to make room for the relative freedom of natural gratification and human drives, the focus is on the enjoyment and pleasure of life and the satisfaction of self-needs (Hofstede et al., 2010). In a cultural context of self-restraint, individuals are more likely to inhibit the satisfaction of their own needs and follow the basic norms of society to discipline their behavior, which is more conducive to transforming their motivation for public service into dedication to their work and society.

### 5.3. Research significance and limitations

In sum, this meta-analysis of the relationship between public service motivation and work engagement clarifies previous research disagreements and confirms the existence of a moderate positive correlation between them, which contributes to the public service literature and work engagement literature. In addition, it also provides theoretical guidance for strategic organizational management in the public sector. It is necessary to recruit civil servants with a higher public service motivation into the public organization, because these people tend to have higher levels of dedication, which may lead to higher performance and administrative efficiency in the public sector. Furthermore, introducing culture as a moderating variable provides a new perspective to understand better the cultural differences between public service motivation and work engagement under different cultural dimensions. It also implies that management practices that reinforce national cultural values are more likely to generate high levels of work engagement in the public service in future public human resource management.

Although the procedures and rules of meta-analysis are strictly followed to analyze the relationship between public service motivation and work engagement from a new perspective, there are still certain shortcomings. First, this paper mainly focuses on Chinese and English articles, and other languages are not included in the analysis, which limits the sample size. Second, only the Hofstede cultural model is used for moderating effects test, but the model has received some criticism, such as involving fewer female groups. In the future, other cultural models can be drawn on for more reliable cross-cultural research. Again, the sample of studies on the relationship between specific dimensions of public service motivation and work engagement is still relatively small, which may lead to insufficient data to support the findings. Therefore, future research needs to focus on each dimension of public service motivation. Finally, this paper only confirms the correlation between public service motivation and work engagement through meta-analysis, but fails to clarify the causal mechanism between them, so future experimental studies are needed to clarify the causal relationship.

## 6. Conclusion

In this paper, a meta-analysis of 31 independent samples reveals a moderate positive relationship between public service motivation and work engagement, which is consistent with the findings of most previous scholars and provides a valuable reference for subsequent research on the relationship between them. The analysis of the four dimensions of public service motivation revealed that the strength of the relationship between attraction to policy making, compassion, commitment to the public interest, self-sacrifice, and work engagement increased in descending order, implying that the impact of each dimension of public service motivation on work engagement varies and needs to be studied more thoroughly in the future. Integrating the results of the moderating effect of cultural dimensions in different countries, it can be concluded that the correlation between public service motivation and work engagement varies across cultural contexts. This study not only broadens the theoretical perspective of previous research on public service engagement but also clarifies the important moderating role of cultural context.

## Data availability statement

The original contributions presented in the study are included in the article/Supplementary material, further inquiries can be directed to the corresponding author.

## Author contributions

MD conceived and designed the study and contributed to the data curation, software, and formal analysis. CW gave suggestions for revision. All authors approved the final manuscript to be published.

## References

[B1] Abdelmotaleb M. (2020). The moderating and mediating role of public service motivation between organization's social responsibility and employee engagement: evidence from egyptian public hospitals. Int. Rev. Public Administr. 25, 207–223. 10.1080/12294659.2020.1815958

[B2] Abdelmotaleb M. Saha S. K. (2018). Corporate social responsibility, public service motivation and organizational citizenship behavior in the public sector. Int. J. Public Administr. 42, 929–939. 10.1080/01900692.2018.1523189

[B3] Akingbola K. Van Den Berg H. A. (2019). Antecedents, consequences, and context of employee engagement in nonprofit organizations. Rev. Public Pers. Administr. 39, 46–74. 10.1177/0734371X16684910

[B4] Andersen L. B. (2009). What determines the behaviour and performance of health professionals? Public service motivation, professional norms and/or economic incentives. Int. Rev. Administr. Sci. 75, 79–97. 10.1177/0020852308099507

[B5] Andersen L. B. Kjeldsen A. M. (2013). Public service motivation, user orientation, and job satisfaction: a question of employment sector? Int. Public Manag. J. 16, 252–274. 10.1080/10967494.2013.817253

[B6] Arcangeli G. Giorgi G. Mucci N. Bernaud J. Di Fabio A. (2018). Editorial: emerging and re-emerging organizational features, work transitions, and occupational risk factors: the good, the bad, the right. An interdisciplinary perspective. Front. Psychol. 9, 1533. 10.3389/fpsyg.2018.01533 30190697 PMC6116519

[B7] Arya S. Schwartz T. A. Ghaferi A. A. (2020). Practical meta-analysis. JAMA Surg. 155, 430–431. 10.1001/jamasurg.2019.4523 31995161

[B8] Auh S. Menguc B. Spyropoulou S. Wang F. (2016). Service employee burnout and engagement: the moderating role of power distance orientation. J. Acad. Market. Sci. 44, 726–745. 10.1007/s11747-015-0463-4

[B9] Bailey C. Madden A. Alfes K. Fletcher L. (2017). The meaning, antecedents and outcomes of employee engagement: a narrative synthesis. Int. J. Manag. Rev. 19, 31–53. 10.1111/ijmr.12077

[B10] Bakker A. B. (2015). A job demands-resources approach to public service motivation. Public Adm. Rev. 75, 723–732. 10.1111/puar.12388

[B11] Bakker A. B. Demerouti E. (2007). The job demands-resources model: state of the art. J. Manag. Psychol. 22, 309–328. 10.1108/0268394071073311531861812

[B12] Bakker A. B. Geurts S. A. E. (2004). Toward a dual-process model of work-home interference. Work Occup. 31, 345–366. 10.1177/0730888404266349

[B13] Bakker A. B. Hakanen J. J. Demerouti E. Xanthopoulou D. (2007). Job resources boost work engagement, particularly when job demands are high. J. Educ. Psychol. 99, 274–284. 10.1037/0022-0663.99.2.27428150993

[B14] Bangert-Drowns R. L. (1986). Review of developments in meta-analytic method. Psychol. Bull. 99, 388–399. 10.1037/0033-2909.99.3.388

[B15] Bashir M. Wright B. E. Hassan S. (2022). The interactive influence of public service motivation, perceived reward equity, and prosocial impact on employee engagement: a panel study in Pakistan. Public Manag. Rev. 1–25. 10.1080/14719037.2021.2013069

[B16] Bland J. T. Williams A. M. Albertson N. (2021). Job-fit and high-performance versus high-empowerment HR: moderators of the PSM—organizational commitment relationship. Public Manag. Rev. 1–26. 10.1080/14719037.2021.1985317

[B17] Borst R. T. (2018). Comparing work engagement in people-changing and people-processing service providers: a mediation model with red tape, autonomy, dimensions of PSM, and performance. Public Pers. Manag. 47, 287–313. 10.1177/0091026018770225 30135612 PMC6088520

[B18] Borst R. T. Kruyen P. M. Lako C. J. (2017). Exploring the job demands–resources model of work engagement in government: bringing in a psychological perspective. Rev. Public Pers. Administr. 39, 372–397. 10.1177/0734371X17729870

[B19] Borst R. T. Kruyen P. M. Lako C. J. de Vries M. S. (2020). The attitudinal, behavioral, and performance outcomes of work engagement: a comparative meta-analysis across the public, semipublic, and private sector. Rev. Public Pers. Administr. 40, 613–640. 10.1177/0734371X19840399

[B20] Boyd N. M. Nowell B. (2020). Sense of community, sense of community responsibility, organizational commitment and identification, and public service motivation: a simultaneous test of affective states on employee well-being and engagement in a public service work context. Public Manag. Rev. 22, 1024–1050. 10.1080/14719037.2020.1740301

[B21] Bright L. (2013). Where does public service motivation count the most in government work environments? A preliminary empirical investigation and hypotheses. Public Pers. Manag. 42, 5–26. 10.1177/0091026013484575

[B22] Britt T. W. Adler A. B. Bartone P. T. (2001). Deriving benefits from stressful events: the role of engagement in meaningful work and hardiness. J. Occup. Health Psychol. 6, 53–63. 10.1037/1076-8998.6.1.53 11199257

[B23] Buchanan B. (1975). Red-tape and the service ethic: some unexpected differences between public and private managers. Administr. Soc. 6, 423–444. 10.1177/009539977500600403

[B24] Cerase F. P. Farinella D. (2009). Public service motivation. Public Policy Administr. 24, 281–308. 10.1177/0952076709103812

[B25] Chen B. Vansteenkiste M. Beyers W. Boone L. Deci E. L. Van der Kaap-Deeder J. . (2015). Basic psychological need satisfaction, need frustration, and nneed strength across four cultures. Motiv. Emot. 39, 216–236. 10.1007/s11031-014-9450-1

[B26] Christopher H. Guy P. (2004). The middle aging of new public management: into the age of paradox? J. Public Administr. Res. Theory 14, 267–282. 10.1093/jopart/muh019

[B27] Clugston M. Howell J. P. Dorfman P. W. (2000). Does cultural socialization predict multiple bases and foci of commitment? J. Manag. 26, 5–30. 10.1177/014920630002600106

[B28] Cooke D. K. Brant K. K. Woods J. M. (2019). The role of public service motivation in employee work engagement: a test of the job demands-resources model. Int. J. Public Administr. 42, 765–775. 10.1080/01900692.2018.1517265

[B29] Crewson P. E. (1997). Public-service motivation: building empirical evidence of incidence and effect. J. Public Administr. Res. Theory 7, 499–518. 10.1093/oxfordjournals.jpart.a024363

[B30] Dawes C. T. Settle J. E. Loewen P. J. Mcgue M. Iacono W. G. (2015). Genes, psychological traits and civic engagement. Philos. Trans. R. Soc. B Biol. Sci. 370, 20150015. 10.1098/rstb.2015.0015 26503688 PMC4633851

[B31] De Dreu C. K. Weingart L. R. Kwon S. (2000). Influence of social motives on integrative negotiation: a meta-analytic review and test of two theories. J. Pers. Soc. Psychol. 78, 173–186. 10.1037/0022-3514.78.5.889 10821196

[B32] Demerouti E. Bakker A. B. (2011). The job demands–resources model: challenges for future research. Sa J. Indus. Psychol. 37, a974. 10.4102/sajip.v37i2.974

[B33] Drory A. Shamir B. (1988). Effects of organizational and life variables on job satisfaction and burnout. Group Organiz. Manag. 13, 441–455. 10.1177/105960118801300403

[B34] Eldor L. Harpaz I. (2019). The nature of learning climate in public administration: a cross-sectorial examination of its relationship with employee job involvement, proactivity, and creativity. Am. Rev. Public Administr. 49, 425–440. 10.1177/0275074018804667

[B35] Fang J. Yu S. Wu Y. Liang J. (2020). The impact of organizational commitment on the job involvement of civil servants: taking public service motivation as mediator variable—an empirical study based on a district-level government in X City. J. South China Univ. Technol. 22, 112–123. 10.19366/j.cnki.1009-055X.2020.02.012

[B36] Frank S. A. Lewis G. B. (2004). Government employees. Am. Rev. Public Administr. 34, 36–51. 10.1177/0275074003258823

[B37] Gagné M. Deci E. L. (2005). Self-determination theory and work motivation. J. Organ. Behav. 26, 331–362. 10.1002/job.322

[B38] Geyfman V. (2014). Engaging government employees: motivate and inspire your people to achieve superior performance. J. Appl. Manag. Entrepr. 19, 121–123. 10.9774/GLEAF.3709.2014.ja.00010

[B39] Giauque D. Ritz A. Varone F. Anderfuhren-Biget S. Waldner C. (2011). Putting public service motivation into context: a balance between universalism and particularism. Int. Rev. Administr. Sci. 77, 227–253. 10.1177/0020852311399232

[B40] Gross H. P. Thaler J. Winter V. (2019). Integrating public service motivation in the job-demands-resources model: an empirical analysis to explain employees' performance, absenteeism, and presenteeism. Int. Public Manag. J. 22, 176–206. 10.1080/10967494.2018.1541829

[B41] Harari M. B. Herst D. E. L. Parola H. R. Carmona B. P. (2016). Organizational correlates of public service motivation: a meta-analysis of two decades of empirical research. J. Public Admin. Res. Theor. 27, 68–84. 10.1093/jopart/muw056

[B42] Harter J. K. Schmidt F. L. Hayes T. L. (2002). Business-unit-level relationship between employee satisfaction, employee eengagement, and business outcomes: a meta-analysis. J. Appl. Psychol. 87, 268–279. 10.1037/0021-9010.87.2.268 12002955

[B43] Higgins J. Thompson S. Deeks J. Altman D. (2002). Statistical heterogeneity in systematic reviews of clinical trials: a critical appraisal of guidelines and practice. J. Health Serv. Res. Policy 7, 51–61. 10.1258/1355819021927674 11822262

[B44] Hobfoll S. E. Johnson R. J. Ennis N. Jackson A. P. (2003). Resource loss, resource gain, and emotional outcomes among inner city women. J. Pers. Soc. Psychol. 84, 632–643. 10.1037/0022-3514.84.3.632 12635922

[B45] Hofstede G. (1984). Culture's Consequences: International Differences in Work-Related Values, Vol. 5. Beverly Hills: Sage.

[B46] Hofstede G. (1991). Cultures Consequences: International Differences in Work Related Values. Beverly Hills, CA: Sage.

[B47] Hofstede G. (2006). What did GLOBE really measure? Researchers' minds versus respondents' minds. J. Int. Business Stud. 37, 882–896. 10.1057/palgrave.jibs.8400233

[B48] Hofstede G. Hofstede G. J. Minkov M. (2010). Cultures and Organizations: Software of the Mind: International Cooperation and its Importance for Survival. New York, NY: McGraw-Hill.

[B49] Hofstede G. Mcrae R. (2004). Personality and culture revisited: linking traits and dimensions of culture. Cross Cult. Res. 38, 52–88. 10.1177/1069397103259443

[B50] Homberg F. Mccarthy D. Tabvuma V. (2015). A meta-analysis of the relationship between public service motivation and job satisfaction. Public Adm. Rev. 75, 711–722. 10.1111/puar.12423

[B51] Homberg F. Vogel R. Weiherl J. (2017). Public service motivation and continuous organizational change: taking charge behaviour at police services. Public Adm. 97, 28–47. 10.1111/padm.12354

[B52] House R. J. Hanges P. J. Javidan M. Dorfman P. W. Gupta V. (Eds.). (2004). Culture, Leadership, and Organizations: The GLOBE Study of 62 Societies. Thousand Oaks: Sage publications.

[B53] Hunter J. E. Schmidt F. L. (2004). Methods of Meta-Analysis: Correcting Error and Bias in Research Findings. Thousand Oaks: Sage.

[B54] Ironson G. H. Smith P. C. Brannick M. T. Gibson W. M. Paul K. B. (1989). Construction of a job in general scale: a comparison of global, composite, and specific measures. J. Appl. Psychol. 74, 193–200. 10.1037/0021-9010.74.2.193

[B55] Jackson C. L. Colquitt J. A. Wesson M. J. Zapata-Phelan C. P. (2006). Psychological collectivism: a measurement validation and linkage to group member performance. J. Appl. Psychol. 91, 884. 10.1037/0021-9010.91.4.884 16834512

[B56] Jensen U. T. Andersen L. B. Holten A. (2018). Explaining a dark side: public service motivation, presenteeism, and absenteeism. Rev. Public Pers. Administr. 39, 487–510. 10.1177/0734371X17744865

[B57] Jeong D. Cho I. Kim K. Lee J. Choi J. M. Kim J. . (2022). Mediating effect of public service motivation and resilience on the association between work-related stress and work engagement of public workers in the COVID-19 pandemic. Psychiatry Investig. 19, 501–510. 10.30773/pi.2021.0403 35700974 PMC9334805

[B58] Jin M. H. Mcdonald B. (2017). Understanding employee engagement in the public sector: the role of immediate supervisor, perceived organizational support, and learning opportunities. Am. Rev. Public Administr. 47, 881–897. 10.1177/0275074016643817

[B59] Kahn W. A. (1990). Psychological conditions of personal engagement and disengagement at work. Acad. Manag. J. 33, 692–724. 10.2307/256287

[B60] Karen L. N. Stanley D. N. (1996). Culture and congruence: the fit between management practices and national culture. *J. Int. Bus. Stud*. 27, 753–779.11055141

[B61] Kepes S. Banks G. C. Mcdaniel M. Whetzel D. L. (2012). Publication bias in the organizational sciences. Organ. Res. Methods 15, 624–662. 10.1177/1094428112452760

[B62] Kim S. (2017). National culture and public service motivation: investigating the relationship using Hofstede's five cultural dimensions. Int. Rev. Administr. Sci. 83(1_suppl.), 23–40. 10.1177/0020852315596214

[B63] Kim S. Vandenabeele W. Wright B. E. Andersen L. B. Cerase F. P. Christensen R. K. . (2013). Investigating the structure and meaning of public service motivation across populations: developing an international instrument and addressing issues of measurement invariance. J. Public Administr. Res. Theory 23, 79–102. 10.1093/jopart/mus027

[B64] Kjeldsen A. M. (2014). Dynamics of public service motivation: attraction-selection and socialization in the production and regulation of social services. Public Adm. Rev. 74, 101–112. 10.1111/puar.12154

[B65] Kwon K. Kim T. (2020). An integrative literature review of employee engagement and innovative behavior: revisiting the JD-R model. Hum. Resour. Manag. Rev. 30, 100704. 10.1016/j.hrmr.2019.100704

[B66] Lawler E. E. Hall D. T. (1970). Relationship of job characteristics to job involvement, satisfaction, and intrinsic motivation. J. Appl. Psychol. 54, 305–312. 10.1037/h0029692

[B67] Lee H. J. Kim M. Y. Park S. M. Robertson P. J. (2020). Public service motivation and innovation in the korean and chinese public sectors: exploring the role of confucian values and social capital. Int. Public Manag. J. 23, 496–534. 10.1080/10967494.2019.1639570

[B68] Leisink P. Steijn B. (2009). Public service motivation and job performance of public sector employees in the Netherlands. Int. Rev. Administr. Sci. 75, 35–52. 10.1177/002085230809950530135612

[B69] Liu B. Tang N. Zhu X. (2008). Public service motivation and job satisfaction in China. Int. J. Manpow. 29, 684–699. 10.1108/01437720810919297

[B70] Luu T. (2018). Discretionary Hr practices and proactive work behaviour: the mediation role of affective commitment and the moderation roles of PSM and abusive supervision. Public Manag. Rev. 20, 789–823. 10.1080/14719037.2017.1335342

[B71] Luu T. T. (2019). Service-oriented high-performance work systems and service-oriented behaviours in public organizations: the mediating role of work engagement. Public Manag. Rev. 21, 789–816. 10.1080/14719037.2018.1526314

[B72] Lynggaard M. Pedersen M. J. Andersen L. B. (2018). Exploring the context dependency of the PSM–performance relationship. Rev. Public Pers. Administr. 38, 332–354. 10.1177/0734371X1667137136438306

[B73] May D. R. Gilson R. L. Harter L. M. (2004). The psychological conditions of meaningfulness, safety and availability and the engagement of the human spirit at work. J. Occup. Organ. Psychol. 77, 11–37. 10.1348/096317904322915892

[B74] Mendez N. Avellaneda C. N. (2022). Organizational commitment in public servants through civic engagement. Public Administr. 1–17. 10.1111/padm.12840

[B75] Min N. Ki N. Yoon T. (2021). Public service motivation, job satisfaction, and the moderating effect of employment sector: a meta-analysis. Int. Rev. Public Administr. 26, 135–155. 10.1080/12294659.2020.1866272

[B76] Moreau D. Gamble B. (2020). Conducting a meta-analysis in the age of open science: tools, tips, and practical recommendations. Psychol. Methods 27, 426–432. 10.1037/met0000351 32914999

[B77] Mostafa A. M. S. Abed El-Motalib E. A. (2020). Ethical leadership, work meaningfulness, and work engagement in the public sector. Rev. Public Pers. Administr. 40, 112–131. 10.1177/0734371X18790628

[B78] Moynihan D. P. Pandey S. K. (2007). Finding workable levers over work motivation: comparing job satisfaction, job involvement, and organizational commitment. Administr. Soc. 39, 803–832. 10.1177/0095399707305546

[B79] Mussagulova A. (2021). Predictors of work engagement: drawing on job demands–resources theory and public service motivation. Austral. J. Public Administr. 80, 217–238. 10.1111/1467-8500.12449

[B80] Perry J. L. (1996). Measuring public service motivation: an assessment of construct reliability and validity. J. Public Administr. Res. Theory 6, 5–22. 10.1093/oxfordjournals.jpart.a024303

[B81] Perry J. L. (2000). Bringing society in: toward a theory of public-service motivation. J. Public Administr. Res. Theory 10, 471–488. 10.1093/oxfordjournals.jpart.a024277

[B82] Perry J. L. Hondeghem A. Wise L. R. (2010). Revisiting the motivational bases of public service: twenty years of research and an agenda for the future. Public Adm. Rev. 70, 681–690. 10.1111/j.1540-6210.2010.02196.x

[B83] Perry J. L. Wise L. R. (1990). The motivational bases of public service. Public Adm. Rev. 50, 367–373. 10.2307/976618

[B84] Rainey H. G. (1982). Reward preferences among public and private managers: in search of the service ethic. Am. Rev. Public Administr. 16, 288–302. 10.1177/027507408201600402

[B85] Rainey H. G. Steinbauer P. (1999). Galloping elephants: developing elements of a theory of effective government organizations. J. Public Admin Res. Theor. 9, 1–32.

[B86] Rice K. (2009). Statistical meta-analysis with applications. J. Am. Stat. Assoc. 104, 1288–1289.

[B87] Ringquist E. (2013). Meta-Analysis for Public Management and Policy. Washington, DC: John Wiley and Sons.

[B88] Ritz A. (2011). Attraction to public policy making: a qualitative inquiry into improvements in PSM measurement. Public Adm. 89, 1128–1147. 10.1111/j.1467-9299.2011.01923.x

[B89] Rosenthal R. (1979). The file drawer problem and tolerance for null results. Psychol. Bull. 86, 638–641. 10.1037/0033-2909.86.3.638

[B90] Ryan R. M. Deci E. L. (2000). Intrinsic and extrinsic motivations: classic definitions and new directions. Contemp. Educ. Psychol. 25, 54–67. 10.1006/ceps.1999.1020 10620381

[B91] Schaufeli W. B. (2002). The measurement of engagement and burnout: a two sample confirmatory factor analytic approach. J. Happiness Stud. 3, 71–92. 10.1023/A:1015630930326

[B92] Schaufeli W. B. (2018). Work engagement in Europe. Organ. Dyn. 47, 99–106. 10.1016/j.orgdyn.2018.01.003

[B93] Schaufeli W. B. Bakker A. B. Salanova M. (2006). The measurement of work engagement with a short questionnaire. Educ. Psychol. Meas. 66, 701–716. 10.1177/0013164405282471

[B94] Schaufeli W. B. Bakker A. B. Van Rhenen W. (2009). How Changes in job demands and resources predict burnout, work engagement, and sickness absenteeism. J. Organ. Behav. 30, 893–917. 10.1002/job.595

[B95] Schaufeli W. B. Taris T. W. van Rhenen W. (2008). Workaholism, burnout, and work engagement: three of a kind or three different kinds of employee well-being? Appl. Psychol. 57, 173–203. 10.1111/j.1464-0597.2007.00285.x

[B96] Shim D. C. Park H. H. Keum J. Kim S. (2021). Street-level bureaucrats' work engagement: can public managers' servant-leader orientation make a difference? Public Pers. Manage. 50, 307–326. 10.1177/0091026020941043

[B97] Singh R. N. Mohanty R. P. (2011). Performance appraisal satisfaction and organisational commitment: moderating role of employees' cultural values. Int. J. Indian Cult. Business Manag. 4, 272. 10.1504/IJICBM.2011.040166

[B98] Song F. Gilbody S. (1998). Bias in meta-analysis detected by a simple, graphical test. increase in studies of publication bias coincided with increasing uuse of meta-analysis. BMJ 316, 471. 10.1136/bmj.316.7129.469 PMC2665616 9492690

[B99] Sun J. Y. Gu Q. (2017). For public causes or personal interests? Examining public service motives in the chinese context. Asia Pac. J. Hum. Resour. 55, 476–497. 10.1111/1744-7941.12119

[B100] Taylor J. (2007). The impact of public service motives on work outcomes in australia: a comparative mulit-dimensional analysis. Public Adm. 85, 931–959. 10.1111/j.1467-9299.2007.00686.x

[B101] Tensay A. T. Singh M. (2020). The nexus between hrm, employee engagement and organizational performance of federal public service organizations in Ethiopia. Heliyon 6, e4094. 10.1016/j.heliyon.2020.e04094 32577549 PMC7303557

[B102] Thornton A. Lee P. (2000). Publication bias in meta-analysis: its causes and consequences. J. Clin. Epidemiol. 53, 207–216. 10.1016/S0895-4356(99)00161-4 10729693

[B103] Tioumagneng A. Njifen I. (2020). Employee involvement in the public administrative sector: an exploration of the engagement drivers in cameroon. Int. Rev. Administr. Sci. 86, 765–781. 10.1177/0020852319838037

[B104] Van Loon N. Kjeldsen A. M. Andersen L. B. Vandenabeele W. Leisink P. (2018). Only when the societal impact potential is high? A panel study of the relationship between public service motivation and perceived performance. Rev. Public Pers. Administr. 38, 139–166. 10.1177/0734371X16639111 29780203 PMC5946672

[B105] Van Loon N. M. Vandenabeele W. Leisink P. (2015). On the bright and dark side of public service motivation: the relationship between PSM and employee wellbeing. Public Money Manag. 35, 349–356. 10.1080/09540962.2015.1061171

[B106] Vandenabeele W. (2007). Toward a public administration theory of public service motivation: an institutional approach. Public Manag. Rev. 9, 545–556. 10.1080/14719030701726697

[B107] Vandenabeele W. (2008). Government calling: public service motivation as an element in selecting government as an employer of choice. Public Adm. 86, 1089–1105. 10.1111/j.1467-9299.2008.00728.x

[B108] Vigoda Gadot E. Eldor L. Schohat L. M. (2013). Engage them to public service. Am. Rev. Public Administr. 43, 518–538. 10.1177/0275074012450943

[B109] Vinarski Peretz H. (2020). A view into managers' subjective experiences of public service motivation and work engagement: a qualitative study. Public Manag. Rev. 22, 1090–1118. 10.1080/14719037.2020.1740304

[B110] Wright B. E. Christensen R. K. Pandey S. K. (2013). Measuring public service motivation: exploring the equivalence of existing global measures. Int. Public Manag. J. 16, 197–223. 10.1080/10967494.2013.817242

[B111] Wright T. A. Bonett D. G. (2007). Job satisfaction and psychological well-being as nonadditive predictors of workplace turnover. J. Manage. 33, 141–160. 10.1177/0149206306297582

[B112] Xanthopoulou D. Bakker A. B. Demerouti E. Schaufeli W. B. (2009). Reciprocal relationships between job resources, personal resources, and work engagement. J. Vocat. Behav. 74, 235–244. 10.1016/j.jvb.2008.11.003

[B113] Yan M. Zhu G. Li M. (2012). A research on the effects of government employees' public service motivation on job involvement. J. Public Administr. 5, 122–144. 10.3969/j.issn.1674-2486.2012.01.013

[B114] Zhu C. Wu C. (2016). Public service motivation and organizational performance in Chinese provincial governments. Chin. Manag. Stud. 10, 770–786. 10.1108/CMS-08-2016-0168

