# Supplementary material for: Can public service motivation increase work engagement?—A meta-analysis across cultures
Source: Front Psychol. 2023 Jan 11;13:1060941. doi: 10.3389/fpsyg.2022.1060941 (PMC9874331; doi:10.3389/fpsyg.2022.1060941)
Supplement: Supplementary file 1 [file Data_Sheet_1.docx]

Supplementary materials

31 studies included in the meta-analysis. This table included the specific coding content.

| Author,  Time | r | APP | CPI | COM | SS | k | WE Measure | PSM  Measure | | Country | PDI | IDV | | MAS | UAI | LTO | IVR |
| --- | --- | --- | --- | --- | --- | --- | --- | --- | --- | --- | --- | --- | --- | --- | --- | --- | --- |
| Abdelmotaleb,  2020 | 0.410 |  |  |  |  | 329 | Others | global | Egypt | | Higher | Collectivism | Low | | Higher | Short-term | Lower |
| Bao,  2018 | 0.210 |  |  |  |  | 216 | UWES | composite | China | | Higher | Collectivism | High | | Lower | Long-term | Lower |
| Bashir et al.,  2021 | 0.385 |  |  |  |  | 56 | UWES | global | Pakistan | | Higher | Collectivism | High | | Higher | Long-term | Lower |
| Bland,  2021 | 0.180 |  |  |  |  | 195 | Others | composite | USA | | Lower | Individualism | High | | Lower | Short-term | Higher |
| Borst,  2017 | 0.500 |  |  |  |  | 9465 | UWES | composite | Netherlands | | Lower | Individualism | Low | | Lower | Long-term | Higher |
| Borst,  2018 |  | 0.230 | 0.380 | 0.310 |  | 24334 | UWES | composite | Netherlands | | Lower | Individualism | Low | | Lower | Long-term | Higher |
| Boyd et al.,  2020 | 0.260 |  |  |  |  | 159 | other | global | USA | | Lower | Individualism | High | | Lower | Short-term | Higher |
| Chen,  2018 | 0.470 |  |  |  |  | 303 | UWES | composite | China | | Higher | Collectivism | High | | Lower | Long-term | Lower |
| Cooke et al.,  2018 | 0.080 |  |  |  |  | 388 | UWES | composite | USA | | Lower | Individualism | High | | Lower | Short-term | Higher |
| Crosby,  2014 | 0.310 |  |  |  |  | 669 | UWES | composite | USA | | Lower | Individualism | High | | Lower | Short-term | Higher |
| De Simone et al.,  2016 | 0.338 |  |  |  |  | 137 | UWES | global | Italy | | Higher | Individualism | High | | Higher | Short-term | Lower |
| Eldor et al.,  2018 | 0.240 |  |  |  |  | 227 | UWES | global | USA | | Lower | Individualism | High | | Lower | Short-term | Higher |
| Fang,  2020 |  | 0.350 | 0.397 | 0.278 | 0.372 | 214 | Others | composite | China | | Higher | Collectivism | High | | Lower | Long-term | Lower |
| Gan,  2020 | 0.457 |  |  |  |  | 468 | UWES | global | China | | Higher | H | High | | Lower | Long-term | Lower |
| Jeong et al.,  2022 | 0.500 |  |  |  |  | 300 | UWES | composite | Korea | | Higher | Collectivism | Low | | Higher | Long-term | Lower |
| Kim,  2015 | 0.560 |  |  |  |  | 11716 | Others | composite | Korea | | Higher | Collectivism | Low | | Higher | Long-term | Lower |
| Li,  2022 | 0.413 |  |  |  |  | 436 | Others | global | China | | Higher | Collectivism | High | | Lower | Long-term | Lower |
| Luu,  2018 | 0.330 |  |  |  |  | 960 | UWES | composite | Vietnam | | Higher | Collectivism | Low | | Lower | Long-term | Lower |
| Luu,  2017 | 0.210 |  |  |  |  | 1183 | Others | composite | Vietnam | | Higher | Collectivism | Low | | Lower | Long-term | Lower |
| Meng et al.,  2014 | 0.407 |  |  |  |  | 581 | UWES | composite | China | | Higher | Collectivism | High | | Lower | Long-term | Lower |
| Mussagulova,2020 | 0.466 |  |  |  |  | 218 | UWES | global | Moldova | | Higher | Collectivism | High | | Lower | Long-term | Lower |
| Park et al.,  2015 | 0.130 |  |  |  |  | 790 | Others | composite | USA | | Lower | Individualism | High | | Lower | Short-term | Higher |
| Qi,  2018 | 0.440 |  |  |  |  | 711 | Others | composite | China | | Higher | Collectivism | High | | Lower | Long-term | Lower |
| Shan et al.,  2019 | 0.390 |  |  |  |  | 171 | UWES | global | China | | Higher | Collectivism | High | | Lower | Long-term | Lower |
| Shim et al.,  2020 | 0.280 |  |  |  |  | 416 | Others | composite | Korea | | Higher | Collectivism | Low | | Higher | Long-term | Lower |
| Sun et al.,  2017 | 0.300 |  |  |  |  | 403 | Others | composite | China | | Higher | Collectivism | High | | Lower | Long-term | Lower |
| Tsai et al.,  2016 | 0.290 |  |  |  |  | 842 | Others | global | USA | | Lower | Individualism | High | | Lower | Short-term | Higher |
| Ugaddan et al.,  2017 | 0.357 |  |  |  |  | 13051 | UWES | global | USA | | Lower | Individualism | High | | Lower | Short-term | Higher |
| Wang,  2020 | 0.560 |  |  |  |  | 458 | UWES | global | China | | Higher | Collectivism | High | | Lower | Long-term | Lower |
| Zhu,  2016 | 0.433 | 0.273 | 0.385 | 0.178 | 0.361 | 761 | UWES | composite | China | | Higher | Collectivism | High | | Lower | Long-term | Lower |
| Zhu,  2012 |  | 0.350 | 0.397 | 0.278 | 0.372 | 761 | Others | composite | China | | Higher | Collectivism | High | | Lower | Long-term | Lower |

Note: r=Pearson correlation coefficient; k=Sample size; APP=Attraction to policymaking; CPI=Commitment to public interest; COM=Compassion; SS=Self-sacrifice; WE=Work engagement; PSM=Public service motivation; PDI=Power Distance Index; IDV=Individualism/Collectivism; MAS=Masculinity/Femininity; UAI=Uncertainty Avoidance Index; LTO=Long-term orientation /Short-term orientation; IVR=Indulgence and Restraint.
